# Identification of dynamic gene expression profiles during sequential vaccination with ChAdOx1/BNT162b2 using machine learning methods

**DOI:** 10.3389/fmicb.2023.1138674

**Published:** 2023-03-17

**Authors:** Jing Li, JingXin Ren, HuiPing Liao, Wei Guo, KaiYan Feng, Tao Huang, Yu-Dong Cai

**Affiliations:** ^1^School of Computer Science, Baicheng Normal University, Baicheng, Jilin, China; ^2^School of Life Sciences, Shanghai University, Shanghai, China; ^3^Changping Laboratory, Beijing, China; ^4^Key Laboratory of Stem Cell Biology, Shanghai Jiao Tong University School of Medicine (SJTUSM) and Shanghai Institutes for Biological Sciences (SIBS), Chinese Academy of Sciences (CAS), Shanghai, China; ^5^Department of Computer Science, Guangdong AIB Polytechnic College, Guangzhou, China; ^6^CAS Key Laboratory of Computational Biology, Bio-Med Big Data Center, Shanghai Institute of Nutrition and Health, University of Chinese Academy of Sciences, Chinese Academy of Science, Shanghai, China; ^7^CAS Key Laboratory of Tissue Microenvironment and Tumor, Shanghai Institute of Nutrition and Health, University of Chinese Academy of Sciences, Chinese Academy of Sciences, Shanghai, China

**Keywords:** SARS-CoV-2, vaccination, immune response, machine learning, blood transcriptome

## Abstract

To date, COVID-19 remains a serious global public health problem. Vaccination against SARS-CoV-2 has been adopted by many countries as an effective coping strategy. The strength of the body’s immune response in the face of viral infection correlates with the number of vaccinations and the duration of vaccination. In this study, we aimed to identify specific genes that may trigger and control the immune response to COVID-19 under different vaccination scenarios. A machine learning-based approach was designed to analyze the blood transcriptomes of 161 individuals who were classified into six groups according to the dose and timing of inoculations, including I-D0, I-D2-4, I-D7 (day 0, days 2–4, and day 7 after the first dose of ChAdOx1, respectively) and II-D0, II-D1-4, II-D7-10 (day 0, days 1–4, and days 7–10 after the second dose of BNT162b2, respectively). Each sample was represented by the expression levels of 26,364 genes. The first dose was ChAdOx1, whereas the second dose was mainly BNT162b2 (Only four individuals received a second dose of ChAdOx1). The groups were deemed as labels and genes were considered as features. Several machine learning algorithms were employed to analyze such classification problem. In detail, five feature ranking algorithms (Lasso, LightGBM, MCFS, mRMR, and PFI) were first applied to evaluate the importance of each gene feature, resulting in five feature lists. Then, the lists were put into incremental feature selection method with four classification algorithms to extract essential genes, classification rules and build optimal classifiers. The essential genes, namely, *NRF2*, *RPRD1B*, *NEU3*, *SMC5*, and *TPX2*, have been previously associated with immune response. This study also summarized expression rules that describe different vaccination scenarios to help determine the molecular mechanism of vaccine-induced antiviral immunity.

## 1. Introduction

Coronavirus disease-19 (COVID-19) is a pandemic infectious disease that is currently affecting many people in approximately 200 countries around the world. It is caused by acute respiratory syndrome coronavirus-2 (SARS-CoV-2), a highly pathogenic coronavirus that belongs to the subfamily Coronaviridae. The SARS-CoV-2 genome contains a variety of structural and nonstructural proteins. The rapid rate at which the virus mutates and spreads has created enormous challenges for prevention and control efforts. Currently, vaccination against SARS-CoV-2 is accepted as an effective strategy against COVID-19 (Folegatti et al., 2020; Amano et al., 2022), with two or more doses giving better protection than one dose alone. The risk of death from COVID-19 varies widely in different countries and may be related to factors such as vaccination rate and number of vaccinations (Masic et al., 2020).

When the body receives the first dose of the COVID-19 vaccine (basic immunization injection), it recognizes viral-specific antigens and produces antibodies and memory cells against SARS-CoV-2. However, the amount of antibodies produced by the primary immune response is much lower than the level required to resist viral invasion. Early clinical trials showed that with just one dose (initial exposure), the body’s resistance to SARS-CoV-2 is very low at about 50%. Therefore, a second vaccine dose and a booster shot have been recommended after a period of time (3–4 weeks). When exposed to the same antigen twice, the memory cells that have been generated in the human body respond rapidly, producing sufficient antibodies and a strong secondary immune response. Therefore, two doses of vaccination are more effective for protection. The ChAdOx1 nCoV-19 (AZD1222) vaccine is constructed from a replication-defective simian adenovirus vector encoding the spike (S) protein of SARS-CoV-2. Clinical trials have shown that the ChAdOx1 vaccine is 74% protective against symptomatic COVID-19 (Cross et al., 2003). Meanwhile, BNT162b2, also known as the Pfizer-BioNTech COVID-19 vaccine, is a messenger RNA (mRNA) vaccine that has been approved by the US FDA for the prevention of COVID-19 caused by the SARS-CoV-2 Beta coronavirus. A heterologous ChAdOx1-S-nCoV-19 and BNT162b2 vaccination combination provides better protection against severe SARS-CoV-2 infection in a real-world observational study (*n* = 13,121). Studies have shown that T-cell responses following ChAdOx1 vaccination were higher than those elicited by BNT162b2. Meanwhile, T-cell responses elicited by BNT162b2 booster doses were enhanced in different vaccination strategies. Both homologous and heterologous vaccinations were able to induce progressively increased frequencies of CD4 and CD8 T cells. However, the heterologous combination elicited stronger CD4 T-cell responses; CD8 T-cell responses were also progressively stronger after the booster dose (Pozzetto et al., 2021). The tolerability and safety profile of BNT162b2 at 30 μg administered as a 2-dose regimen are favorable. In participants who received only one ChAdOx1 dose, antibodies against the SARS-CoV-2 spike protein peaked at day 28 (median 157 ELISA units [EU]); on day 56, the median was 119 EU. Among participants who received the booster dose, the median antibody at day 56 was 639 EU (Folegatti et al., 2020). Studies have demonstrated the efficacy of a two-dose regimen of the BNT162b2 vaccine (Mizrahi et al., 2021).

An increasing number of studies have confirmed that high-throughput sequencing data information can provide important guidance for revealing the pathogenic mechanism of diseases and tackling various medical problems (Dai et al., 2018; Kong et al., 2020; Yang et al., 2020, 2022). Our team has long been working on using machine learning analysis methods to screen for disease-related signatures and explain their pathogenic mechanisms. We divided the data on 161 people vaccinated against COVID-19 into six groups according to the injection and vaccination time, aiming to further explore changes in blood gene expression after different doses, especially the molecular characteristics of antiviral immunity. A variety of algorithms were used to analyze gene expression information on vaccines from different vaccinations. The algorithms included feature ranking algorithms, such as least absolute shrinkage and selection operator (Lasso) (Tibshirani, 2011), light gradient-boosting machine (LightGBM) (Ke et al., 2017), Monte Carlo feature selection (MCFS) (Dramiński et al., 2007), max-relevance and min-redundancy (mRMR) (Peng et al., 2005), and permutation feature importance (PFI) (Fisher et al., 2019), as well as classification algorithms, such as decision tree (DT) (Safavian and Landgrebe, 1991), random forest (RF) (Breiman, 2001), K-nearest neighbor (KNN) (Cover and Hart, 1967), and support vector machine (SVM) (Cortes and Vapnik, 1995). Based on feature ranking algorithms, gene feature lists were obtained, which were subjected to incremental feature selection (IFS) method (Liu and Setiono, 1998), incorporating four classification algorithms, for extracting essential genes, classification rules, and build optimal classifiers. This study revealed that blood gene expression changed after the initial immunization and booster vaccination. A number of important genes (e.g., *NRF2*, *RPRD1B*, *NEU3*, *SMC5*, and *TPX2*) may be closely related to the antiviral immunity induced by vaccines. These findings are helpful for understanding the importance of vaccination and boosting injections by revealing the effects of different injections on the expression of immune-related molecules in the host and by providing a reference for viral immune intervention strategies for COVID-19.

## 2. Materials and methods

**Figure 1:**
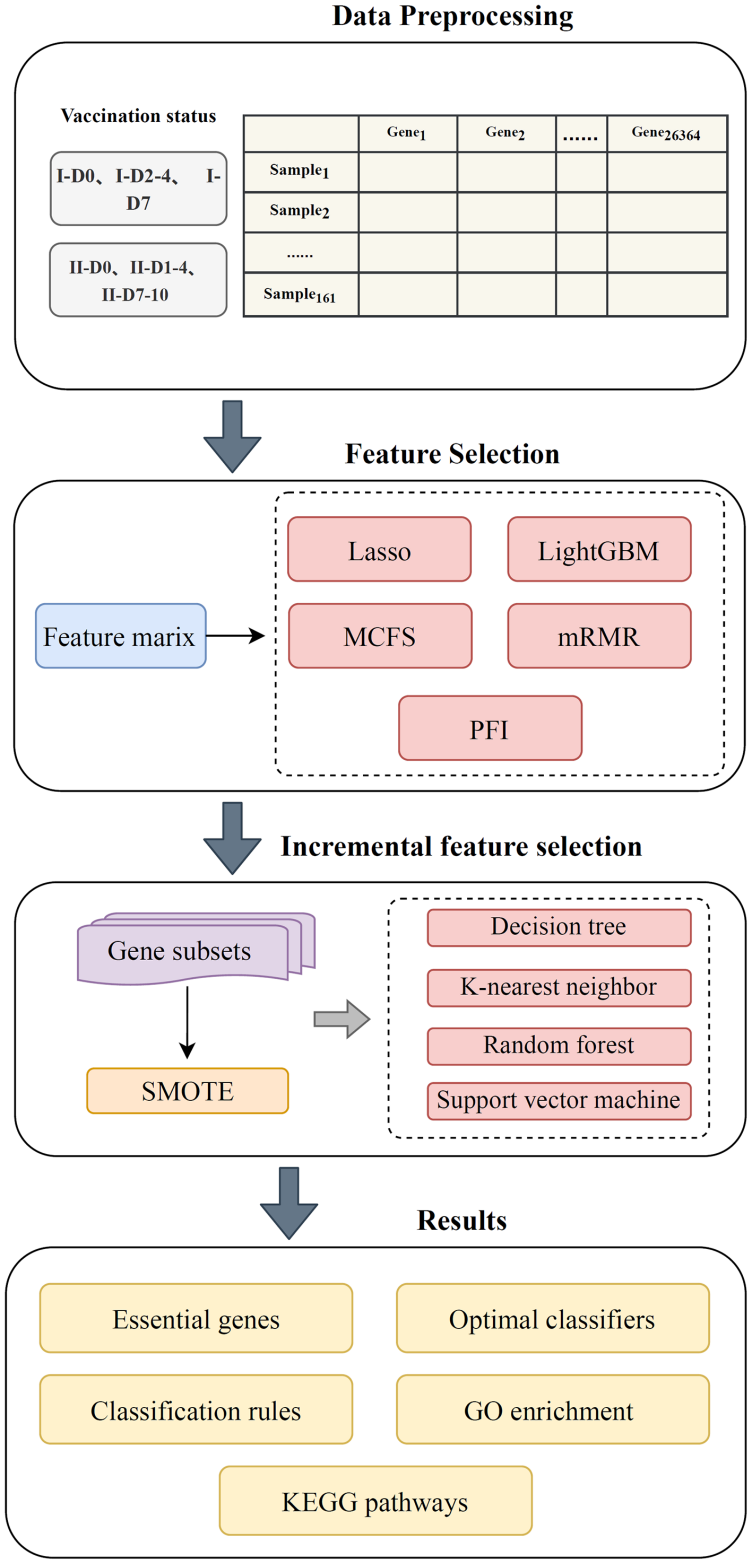
Flow chart of the entire analysis process. The blood transcriptome data of 161 vaccinees with different COVID-19 vaccination was investigated. Each vaccinee was represented by 26,364 gene expression levels. Five feature ranking algorithms (Lasso, LightGBM, MCFS, mRMR, and PFI) were used to rank gene features according to their importance. Subsequently, these lists were fed into incremental feature selection method, which contained four classification algorithms, to extract essential genes, classification rules, and build optimal classifiers.

The workflow of the machine learning framework is shown in Figure 1. The samples were grouped according to the number of inoculations and inoculation time. The genes were subsequently ranked using five methods and further processed by IFS method with four classification algorithms. By observing the performance of the classifiers, a number of key genes and summarized quantitative classification rules were identified. Last, the key genes were functionally enriched to determine the biological processes involved in their action. The methods used are described in detail in this section.

### 2.1. Data

Blood transcriptome data from 161 individuals were obtained from the GEO database under the registration number GSE201533 (Lee et al., 2022a). We divided the vaccinees into two groups: I for the first COVID-19 vaccination dose and II for the second dose. For the first group, three subsets were included: I-D0, I-D2-4, and I-D7, meaning day 0, days 2–4, and day 7 after the first dose of ChAdOx1, respectively. There were also three subsets in the second group, say II-D0, II-D1-4, II-D7-10, meaning the day 0, days 1–4, and days 7–10 after the second dose of BNT162b2, respectively. Four of the vaccinees received a second dose of ChAdOx1. Table 1 shows the number of samples in each subset. Each sample was represented by 26,364 gene expression levels, which were deemed as features in this study. The six subsets (I-D0, I-D2-4, I-D7, II-D0, II-D1-4, and II-D7-10) were termed as labels. The current study was conducted by deeply investigating such classification problem.

**Table 1:** Sample sizes of six vaccination status.

Index	Vaccination status		Sample size
1	I-D0	(Day 0 after the first dose)	37
2	I-D2-4	(Day2 2–4 after the first dose)	36
3	I-D7	(Day 7 after the first dose)	37
4	II-D0	(Day 0 after the second dose)	17
5	II-D1-4	(Days 1–4 after the second dose)	18
6	II-D7-10	(Days 7–10 after the second dose)	16

### 2.2. Feature ranking algorithms

Lots of features were used to represent each sample. Evidently, some were important and others were useless. It was necessary to extract important features. To date, several feature analysis methods have been proposed, which can evaluate the importance of features. The selection of such method is a challenge problem as each method has its own merits and defects. Generally, one method can only output a part of essential features. Thus, it was beneficial to employ multiple methods, thereby providing a more complete picture on essential features. Here, five algorithms, namely, Lasso (Tibshirani, 2011), LightGBM (Ke et al., 2017), MCFS (Dramiński et al., 2007), mRMR (Peng et al., 2005), and PFI (Fisher et al., 2019), were employed to rank genes according to their importance. These algorithms have been frequently applied to solve many life science problems (Zhao et al., 2018; Ren et al., 2022; Li et al., 2022a,b,c; Huang et al., 2023a,b).

#### 2.2.1. Least absolute shrinkage and selection operator

Based on the nonnegative garrote proposed by Breiman (1995), Robert Tibshirani first proposed the Lasso algorithm in 1996 (Tibshirani, 2011). The algorithm proposes a first-order penalty function containing regularized formulas, where each feature is regarded as an independent variable in the function. The coefficients of the features are then obtained by solving the optimization function. The absolute value of a coefficient indicates the degree of correlation of each feature to the target dependent variable. To achieve data compression and reduce overfitting, the algorithm regularizes the coefficients of some variables while setting some to zero to eliminate the features that tend to contribute less to the follow-up prediction. Accordingly, the algorithm can rank features according to the absolute values of their coefficients. In present study, the Lasso program in Scikit-learn (Pedregosa et al., 2011) was adopted, which was executed using default parameters.

#### 2.2.2. Light gradient-boosting machine

LightGBM (Ke et al., 2017) is based on the gradient-boosting decision tree framework and introduces gradient one-sided sampling, exclusive feature bundling, histogram algorithm, and leaf-wise growth strategy. It enables data slicing, bundling, and dimensionality reduction and ultimately reduces computational cost while improving prediction accuracy. The importance of each feature is determined by the number of trees that the feature participates in building: the higher the participation, the higher the importance. Thus, features can be ranked in a list with decreasing order of this number. The current study used the LightGBM program obtained from.^1^ For convenience, it was performed using default parameters.

#### 2.2.3. Monte Carlo feature selection

Monte Carlo feature selection was originally developed by Dramiński et al. (2007). The algorithm selects some features randomly and repeatedly to obtain *p* feature subsets. Each feature subset is then divided into a training set and a test set t times, and t trees are constructed. Thus, *p* × *t* trees are obtained. The importance of features can be evaluated by their contributions to building these trees and is defined as the relative importance (RI) score, which is calculated as follows:

(1) $$RI_{g}=\overset{p\times t}{\underset{\tau =1}{\sum }}{\left(\omega A_{CC}\right)}^{u}\underset{ng\left(\tau \right)}{\sum }IG\left(ng\left(\tau \right)\right){\left(\frac{no.in\;ng\left(\tau \right)}{no.in\;\tau }\right)}^{v},$$

where $\omega A_{CC}$ is the weighted precision of the tree τ under consideration, ng(τ) is a node of the tree whose information gain is denoted as IG(ng(τ)) , and no.inng(τ)(no.inτ) denotes the sample size of ng(τ)(τ) . u and v are two positive numbers weighting the $\omega A_{CC}$ and the ratio no.inng(τ)/no.inτ , respectively. To execute MCFS, we downloaded its program from.^2^ Default parameters were used.

#### 2.2.4. Max-relevance and Min-redundancy

The mRMR method was proposed by Peng et al. (2005) in 2005. It screens features based on their correlation with the target variable and the redundancy between features. The correlation and redundancy can be calculated from the mutual information between features or target variables. The tradeoff of correlation and redundancy is used to evaluate the importance of features. At each round, one feature with the maximum correlation to target variables and minimum redundancy to features in the current list is selected and appended to the current list. Here, we used the mRMR program sourced from.^3^ It was executed with default parameters.

#### 2.2.5. Permutation feature importance

The PFI for RFs was first introduced in 2001 by Breiman (2001) and was later extended to any fitted estimator for features by Fisher et al. (2019). The idea is relatively simple. If a feature is important, the prediction error will further increase after the feature’s values are shuffled. If a feature is not important, shuffling its values does not increase the prediction error. The PFI program used in this study was retrieved from scikit-learn (Pedregosa et al., 2011), which was executed with default parameters.

Above five algorithms were applied to the blood transcriptome data one by one. Each algorithm produced one feature list. For easy descriptions, the generated lists were called Lasso, LightGBM, MCFS, mRMR and PFI feature lists.

### 2.3. Incremental feature selection

When the feature list contains an excessive number of features, it is not suitable for direct use in building prediction models. In this study, the IFS (Liu and Setiono, 1998) method was used to extract the best subset of features. From the feature list, a series of feature subsets can be constructed. Each subset includes 10 more features than the previous subset in the order of the list. These feature subsets were then fed to one classification algorithm to build the classifier. The performance of these classifiers was evaluated by 10-fold cross-validation. Lastly, the best classifier can be obtained, which was termed as the optimal classifier. The feature subset for constructing this classifier was called the optimal feature subset.

### 2.4. Synthetic minority oversampling technique

According to Table 1, some classes (e.g., I-D0) contained much more samples than other classes (e.g., II-D7-10). The dataset was imbalanced. The results of the classifier would have preferences for the majority class when the number of samples from different categories differs significantly. This study used synthetic minority oversampling technique (SMOTE) (Chawla et al., 2002) to balance the dataset. For each class with a small number of samples, a sample is random chosen. Then its k nearest neighbors in the same class are identified by Euclidean distance. A neighbor is randomly selected. A new sample is then randomly generated by linearly interpolating the randomly chosen sample and the selected nearest neighbor. New samples are continuously generated until such class contains samples as many as those in the largest class. The SMOTE package reported in^4^ was used in this study. Default settings were adopted.

### 2.5. Classification algorithms for building classifiers

Four classification algorithms were used in the IFS approach. Key genes were then screened based on the performance of the constructed classifiers.

#### 2.5.1. Decision tree

The DT algorithm (Safavian and Landgrebe, 1991) constructs a tree-like structure in which instances are judged in each internal node of the tree. Starting from the root node, all samples are assigned to different classes through continuous judgments. Each tree branch contains clues to the classification of instances and thus provides interpretable classification rules that underlie the understanding of biological mechanisms. In this study, we used the CART classification tree algorithm with node ranking using the Gini coefficient.

#### 2.5.2. Random forest

In the RF algorithm for classification, a judgment is completed by constructing DTs based on different training sets and then combining their results to make predictions (Breiman, 2001; Wang et al., 2021; Ran et al., 2022; Tang and Chen, 2022; Wu and Chen, 2023). The training set with the same number of samples in the input dataset is repeatedly sampled to generate numerous new training sets. Each new training set is then used to build a new DT, and an ensemble of DTs is constructed. Given a new instance, each DT makes a prediction. Predictions taken from all DTs are combined to reach a final decision.

#### 2.5.3. K-nearest neighbor

In KNN (Cover and Hart, 1967), new samples are predicted by comparing each with samples with known labels (training samples) and determining the k-nearest neighbors. Subsequently, the class of a new sample is determined by voting according to the classes of the k-nearest neighbors. In this study, the distance was defined as the Minkowski distance.

#### 2.5.4. Support vector machine

The SVM algorithm (Cortes and Vapnik, 1995; Wang and Chen, 2022; Wang and Chen, 2023) utilizes a kernel function that maps the attributes of the instances, i.e., the feature vectors, into a higher-dimensional space and attempts to find a separating hyperplane. This hyperplane partitions the instances by class and ensures that the margin between the two categories is maximum. This method is generally to have good generalization.

We adopted public packages in scikit-learn (Pedregosa et al., 2011) to implement above four classification algorithms. All packages were performed using default parameters.

### 2.6. Performance evaluation

In the multi-class classification problem, weighted F1 is an important measurement to evaluate the performance of the classifier. It is obtained by calculating and integrating the F1-measure values of different classes based on the proportion of the samples in each class. It is known that F1-measure is an integrated measurement combining precision and recall, which can be computed by

(2) $$\mathrm{Pr}ecision_{i}=\frac{TP_{i}}{TP_{i}+FP_{i}},$$

(3) Recalli=TPiTPi+FNi,

(4) $$F1-measure_{i}=\frac{2\times Precision_{i}\times Recall_{i}}{Precision_{i}+Recall_{i}},$$

where *i* represents the index of class, TP represents true positive, FP represents false positive, and FN represents false negative. Then, weighted F1 can be calculated by

(5) $$Weighted\;F1=\overset{L}{\underset{i=1}{\sum }}w_{i}\times F1-measure_{i},$$

where L represents the number of classes and $w_{i}$ represents the proportion of samples in the *i*-th class to overall samples. Here, weighted F1 was selected as the major measurement.

In addition, overall accuracy (ACC) and Matthew correlation coefficient (MCC) (Matthews, 1975) are also widely used to assess the quality of classifiers. ACC is defined as the proportion of correctly predicted samples to all samples. MCC is a balanced measurement, which is more objective than ACC when the dataset is imbalanced. For the calculation of MCC, two matrices *X* and *Y* must be constructed first, which store the one-hot representation of true and predicted class of each sample. Then, MCC can be computed by

(6) $$MCC=\frac{\mathrm{cov}\left(X,Y\right)}{\sqrt{\mathrm{cov}\left(X,X\right)\mathrm{cov}\left(Y,Y\right)}}$$

where cov(X,Y) denotes the correlation coefficient of X  and Y .

### 2.7. Functional enrichment analysis

Using the IFS method, we can obtain the best subset of features under different rankings. To clarify the biological processes behind genes in these subsets, thereby uncovering their relationship with antiviral immunity, this study used gene ontology (GO) enrichment analysis to discover the role of the genes and applied Kyoto Encyclopedia of Genes and Genomes (KEGG) pathway analysis to identify the underlying pathways. ClusterProfiler package (Wu et al., 2021) in R was used to perform GO and KEGG enrichment analyses.

## 3. Results

### 3.1. Results of feature ranking

To evaluate the importance of features from multiple aspects. Five feature ranking algorithms were employed, which were applied to the blood transcriptome data one by one. As a result, five feature lists, named Lasso, LightGBM, MCFS, mRMR and PFI feature lists, were obtained, which are provided in Supplementary Table S1. Table 2 shows the top 10 genes in each list. It can be observed that top genes in different lists were very different, meaning that the importance of one feature was quite different under the evaluation of different methods. Usage of different methods can provide more opportunities to discover more essential features.

**Table 2:** The top 10 features in five feature lists.

Index	Lasso feature list	LightGBM feature list	MCFS feature list	mRMR feature list	PFI feature list
1	CENPF	RPRD1B	NEU3	FAM98B	SLC16A14
2	NDUFB9	ITM2C	C2	TSSK4	THRAP3
3	BRCA2	HSP90B1	SMC5	CSF1R	STAC3
4	LOC102031319	TK1	ZFC3H1	TOP1	ATF5
5	SSBP1	LPAR3	GLS2	NEU3	RAD51
6	PDP1	CENPF	NFE2L2	UBE2H	CDC45
7	LINC01089	TPX2	C1QC	ATP6V1E1	GABPB1
8	C2orf16	ITGAE	SDC1	SRPRB	CTNNBL1
9	ID2	SPATA24	CAV1	ZNF672	ARHGAP42
10	LINC00630	GTSE1	SNORA2B	CUL3	PSME2

### 3.2. Results of incremental feature selection

**Figure 2:**
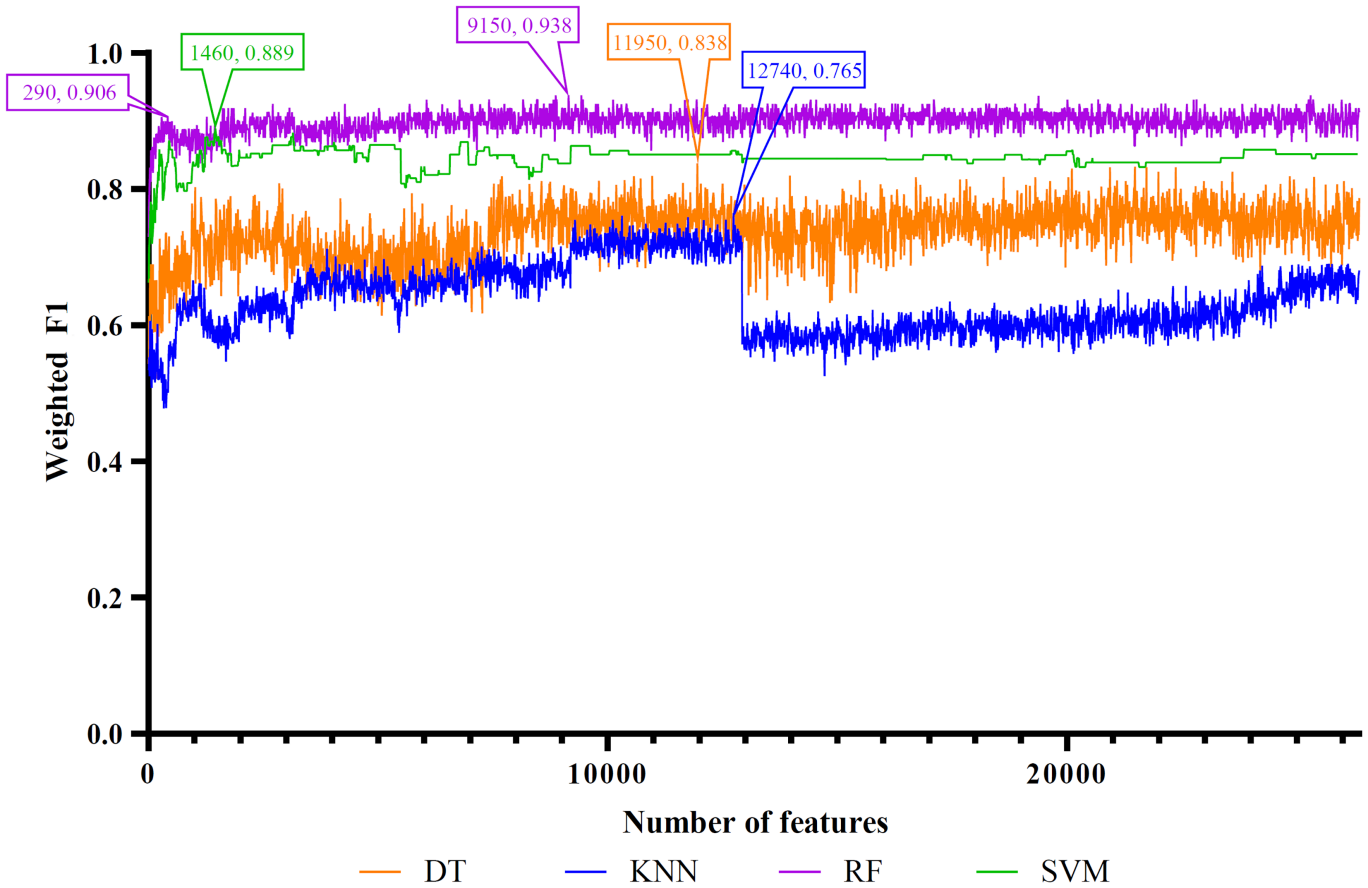
IFS curves of four classification algorithms on Lasso feature list. DT, KNN, RF, and SVM yielded the highest weighted F1 values of 0.838, 0.765, 0.938, and 0.889 when top 11,950, 12,740, 9,150, and 1,460 features were adopted, respectively. RF can yield quite high performance (weighted F1 = 0.906) when top 290 features were used.

**Figure 3:**
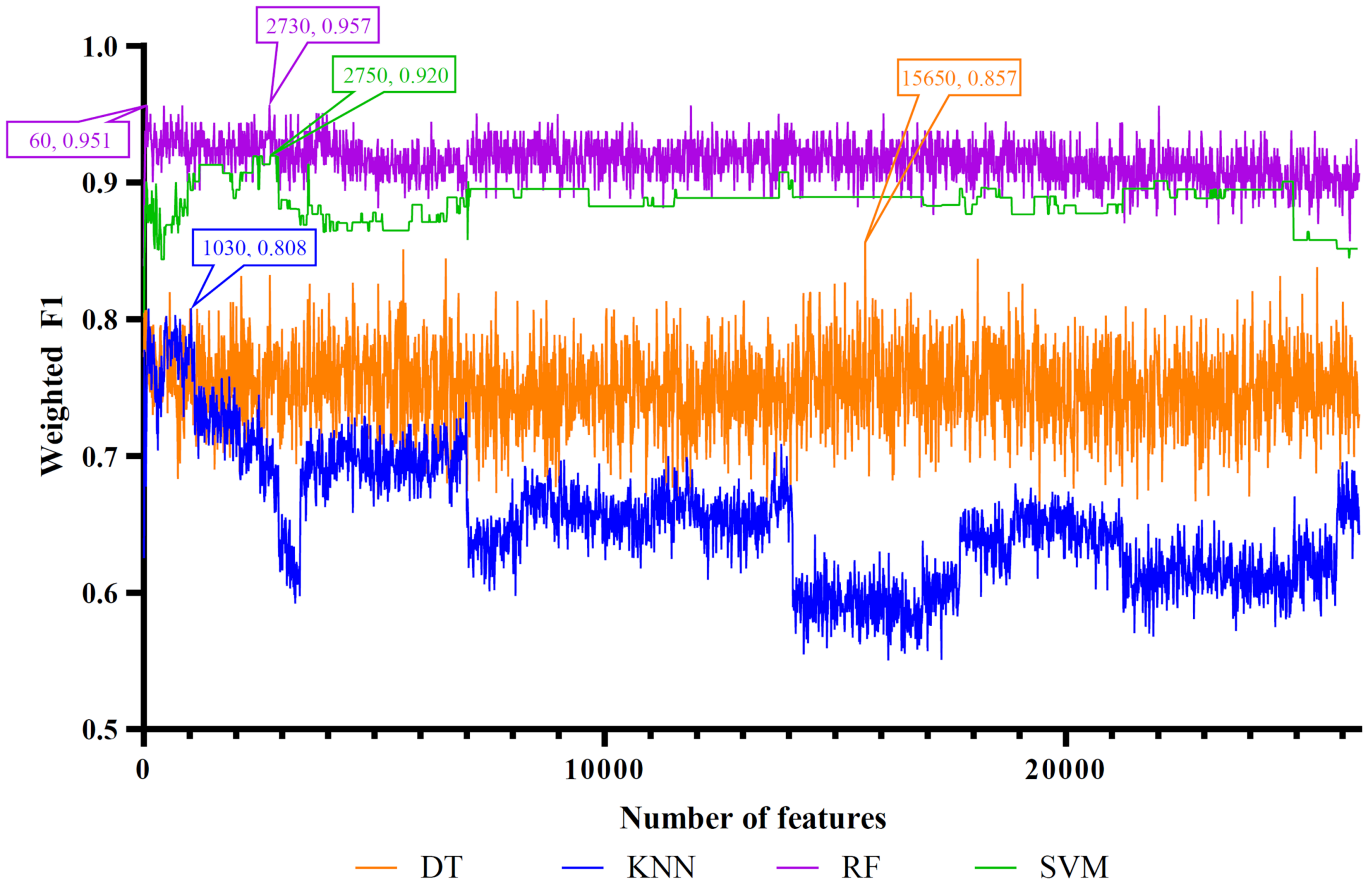
IFS curves of four classification algorithms on LightGBM feature list. DT, KNN, RF, and SVM yielded the highest weighted F1 values of 0.857, 0.808, 0.957, and 0.920 when top 15,650, 1,030, 2,730, and 2,750 features were adopted, respectively. RF can yield quite high performance (weighted F1 = 0.951) when top 60 features were used.

**Figure 4:**
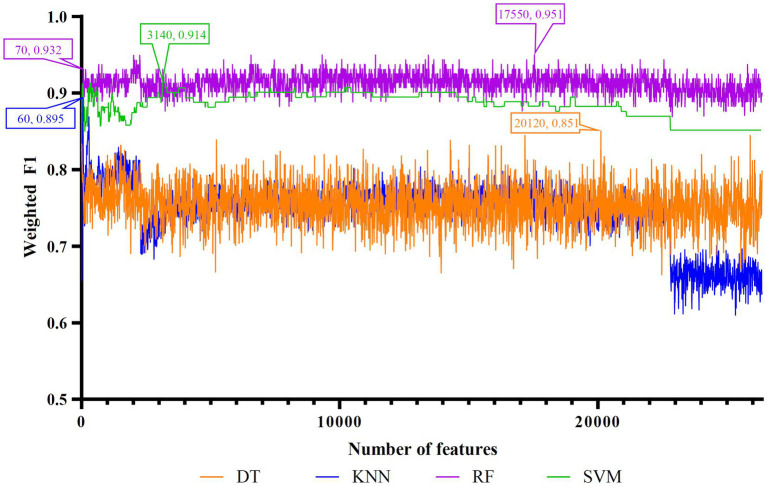
IFS curves of four classification algorithms on MCFS feature list. DT, KNN, RF and SVM yielded the highest weighted F1 values of 0.851, 0.895, 0.951, and 0.914 when top 20,120, 60, 17,550, and 3,140 features were adopted, respectively. RF can yield quite high performance (weighted F1 = 0.932) when top 70 features were used.

**Figure 5:**
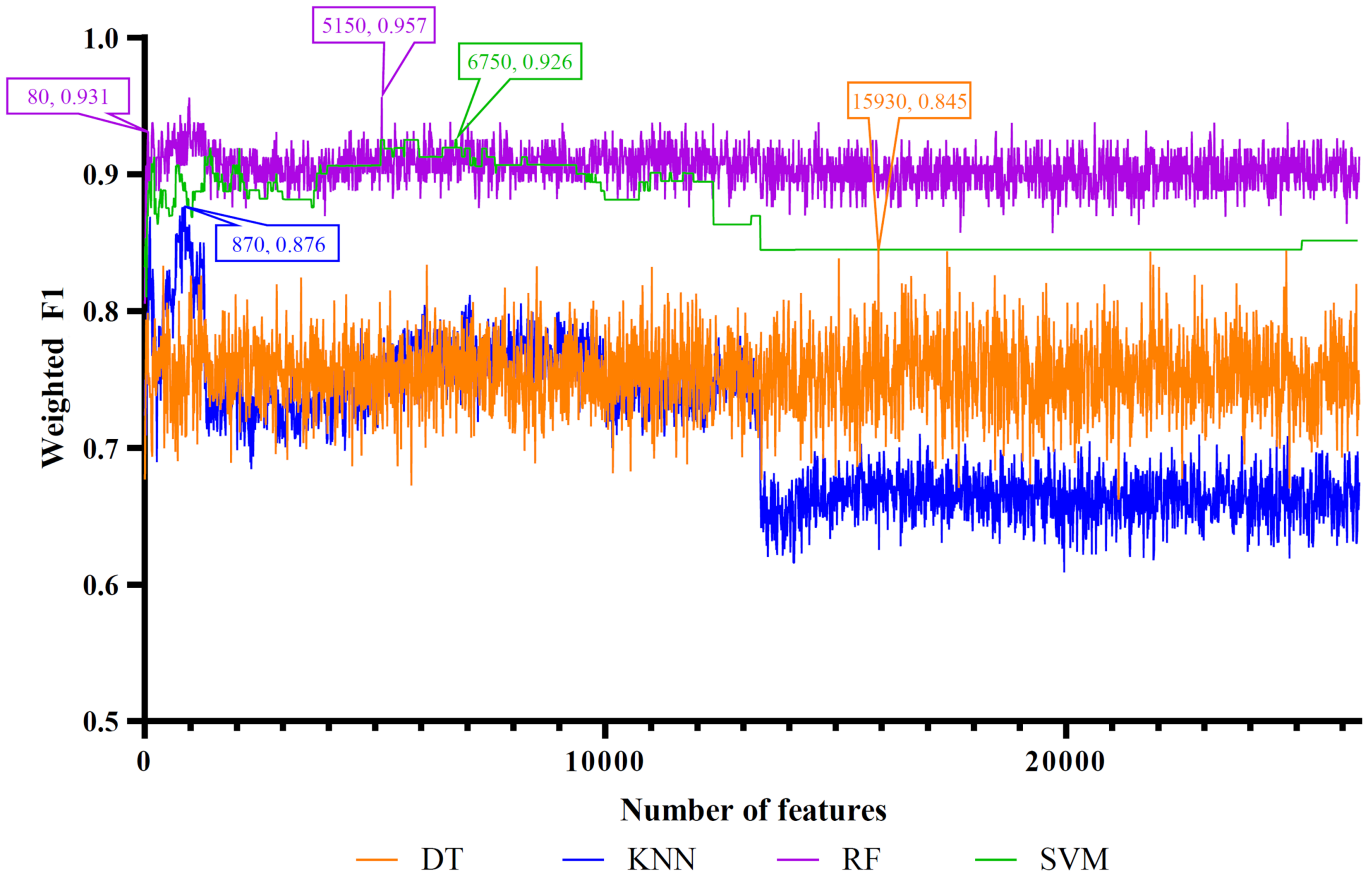
IFS curves of four classification algorithms on mRMR feature list. DT, KNN, RF, and SVM yielded the highest weighted F1 values of 0.845, 0.876, 0.957, and 0.926 when top 15,930, 870, 5,150, and 6,750 features were adopted, respectively. RF can yield quite high performance (weighted F1 = 0.931) when top 80 features were used.

**Figure 6:**
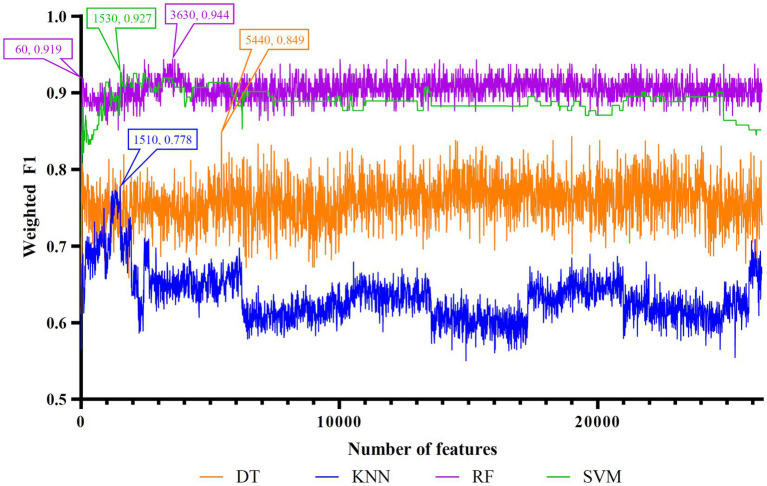
IFS curves of four classification algorithms on PFI feature list. DT, KNN, RF, and SVM yielded the highest weighted F1 values of 0.849, 0.778, 0.944, and 0.927 when top 5,440, 1,510, 3,630, and 1,530 features were adopted, respectively. RF can yield quite high performance (weighted F1 = 0.919) when top 60 features were used.

Five feature lists were subjected to the IFS method one by one. From each feature list, a series of feature subsets with step ten were constructed. On each subset, one classifier was built for each of four classification algorithms (DT, KNN, RF, and SVM). When constructing the classifiers, the dataset was processed by SMOTE to tackle the imbalanced problem. All classifiers were evaluated by 10-fold cross-validation. The evaluation results were counted as weighted F1, ACC, and MCC, which are provided in Supplementary Table S2. Weighted F1was selected as the major measurement. Thus, several IFS curves were plotted for different classification algorithms and feature lists, as shown in Figures 2–6, in which weighted F1 was set as Y-axis and number of features was defined as *X*-axis.

For the Lasso feature list, the IFS curves of four classification algorithms are illustrated in Figure 2. It can be observed that when top 11,950, 12,740, 9,150 and 1,460 features were adopted, four algorithms yielded the highest weighted F1 values of 0.838, 0.765, 0.938, and 0.889, respectively. Thus, the optimal DT, KNN, RF, and SVM classifiers can be built using these features. The ACC and MCC values of these classifiers are listed in Table 3. Evidently, the optimal RF classifier was best among these optimal classifiers.

**Table 3:** Performance of the optimal classifiers based on different classification algorithms and feature lists.

Feature list	Classification algorithm	Number of features	Weighted F1	MCC	ACC
Lasso feature list	Decision tree	11,950	0.838	0.801	0.839
	K-nearest neighbor	12,740	0.765	0.722	0.770
	Random forest	9,150	0.938	0.924	0.938
	Support vector machine	1,460	0.889	0.863	0.888
LightGBM feature list	Decision tree	15,650	0.857	0.825	0.857
	K-nearest neighbor	1,030	0.808	0.764	0.807
	Random forest	2,730	0.957	0.947	0.957
	Support vector machine	2,750	0.920	0.901	0.919
MCFS feature list	Decision tree	20,120	0.851	0.817	0.851
	K-nearest neighbor	60	0.895	0.870	0.894
	Random forest	17,550	0.951	0.939	0.950
	Support vector machine	3,140	0.914	0.894	0.913
mRMR feature list	Decision tree	15,930	0.845	0.809	0.845
	K-nearest neighbor	870	0.876	0.847	0.876
	Random forest	5,150	0.957	0.947	0.957
	Support vector machine	6,750	0.926	0.908	0.925
PFI feature list	Decision tree	5,440	0.849	0.817	0.851
	K-nearest neighbor	1,510	0.778	0.734	0.783
	Random forest	3,630	0.944	0.932	0.944
	Support vector machine	1,530	0.927	0.909	0.925

For the LightGBM feature list, Figure 3 shows the IFS curves of four classification algorithms. The optimal DT/KNN/RF/SVM classifier can be built using top 15,650/1030/2730/2750 features in this list. Their ACC, MCC, and weighted F1 values are listed in Table 3. Clearly, RF still provided the best performance as the optimal RF classifier yielded the highest weighted F1 of 0.957.

As for the rest three feature lists, the IFS curves are shown in Figures 4–6. The optimal DT/KNN/RF/SVM classifier can be set up on each feature list. The numbers of top features used in these classifiers are listed in Table 3, where the performance of these classifiers is also provided. Similar to the results on the Lasso and LightGBM feature lists, the optimal RF classifier was also better than other three optimal classifiers on each feature list.

**Figure 7:**
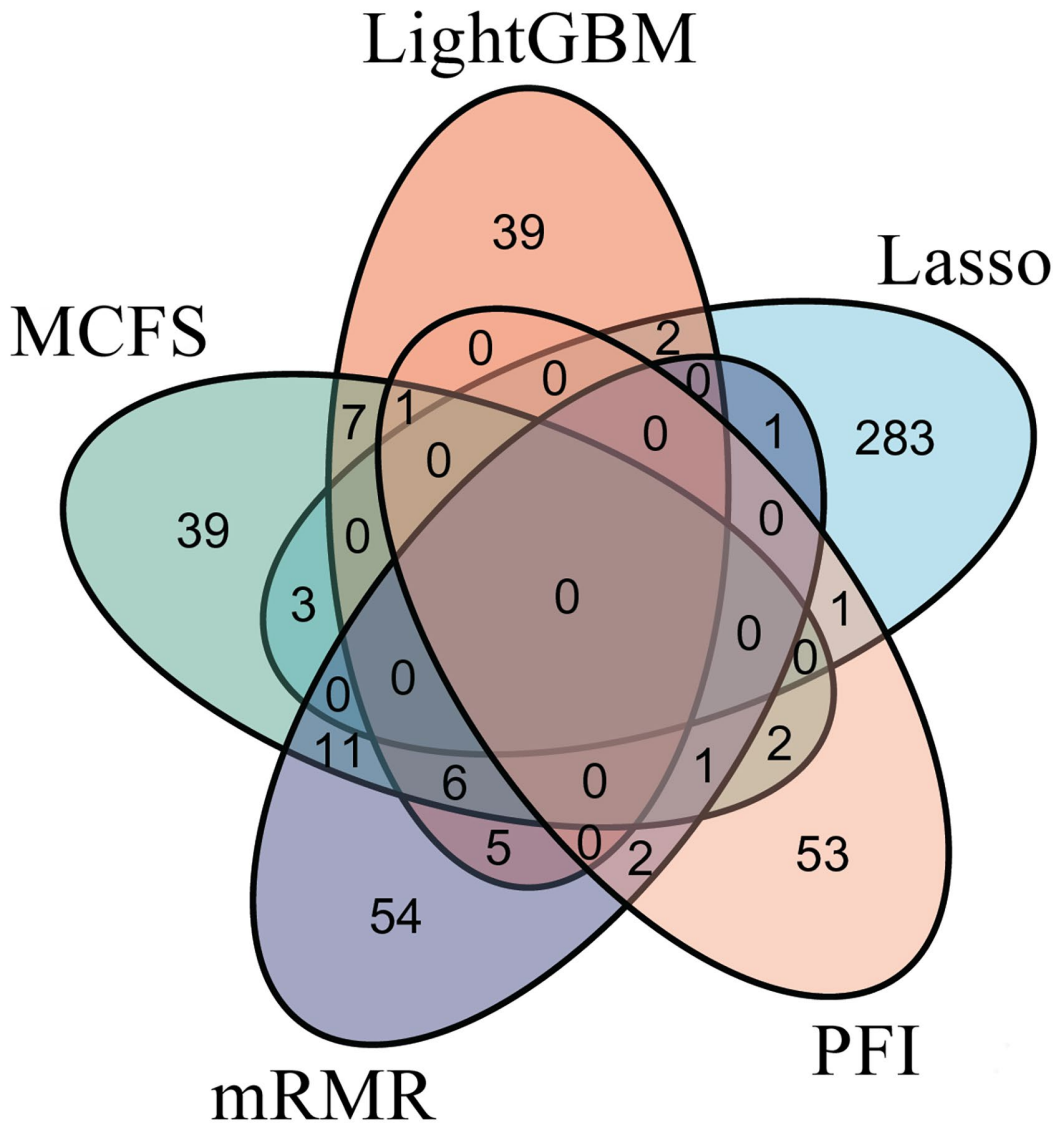
Venn diagram of the feature sets used to construct feasible random forest classifiers on five feature lists that were obtained by Lasso, LightGBM, MCFS, mRMR, and PFI, respectively. The overlapping circles indicated genes that occurred in multiple sets. These genes were deemed to be important by multiple feature ranking algorithms.

To make full use of the utility of five algorithms, the best features should be extracted from each feature list, thereby obtaining the latent essential gene features. As mentioned above, the optimal RF classifier was best for each feature list. Thus, the features used in these classifiers can be picked up as important candidates. However, such feature numbers (9,150 for Lasso feature list, 2,730 for LightGBM feature list, 17,750 for MCFS feature list, 5,150 for mRMR feature list, 3,630 for PFI feature list) were too large to make detailed analyses. In view of this, we tried to find out another RF classifier, which adopted much less features and provided a little lower performance than the optimal RF classifier, on each feature list. By carefully checking the IFS results on RF on each feature list, such RF classifiers adopted the top 290 features in the Lasso feature list, top 60 features in the LightGBM feature list, top 70 features in the MCFS feature list, top 80 features in the mRMR feature list, and top 60 features in the PFI feature list. The corresponding points have been marked on the IFS curves of RF, as illustrated in Figures 2–6. The detailed performance of these RF classifiers is listed in Table 4. It can be observed that their performance was still quite high, the weighted F1 values were all higher than 0.900. Compared with the weighted F1 yielded by the optimal RF classifier on the same feature list, this RF classifier provided a little lower weighted F1. However, their efficiencies were sharply improved because much less features were involved. This indicated the extreme importance of features used in these RF classifiers. For easy descriptions, these RF classifiers were called feasible RF classifiers. Furthermore, the performance of the feasible RF classifier on one feature list was generally better than the optimal DT/KNN/SVM classifier on the same feature list, further confirming the importance of features in the feasible RF classifiers. To clear show the relationship between the feature sets used in five feasible RF classifiers, a Venn diagram was plotted, as shown in Figure 7. The detailed results of the intersection are shown in Supplementary Table S3. Some gene features occurred in multiple subsets, meaning that they were deemed to be important by multiple feature ranking algorithms. They may have strong associations with antiviral immunity. Some of them would be discussed in detail in the subsequent sections.

**Table 4:** Performance of feasible classifiers on different feature list.

Feature list	Classification algorithm	Number of features	Weighted F1	MCC	ACC
Lasso feature list	Random forest	290	0.906	0.886	0.907
LightGBM feature list	Random forest	60	0.951	0.939	0.950
MCFS feature list	Random forest	70	0.932	0.916	0.932
mRMR feature list	Random forest	80	0.931	0.916	0.932
PFI feature list	Random forest	60	0.919	0.901	0.919

### 3.3. Classification rules

Although the performance of DT was much lower than RF and SVM according to the IFS results on five feature lists, DT has an exclusive merit as it is a white-box algorithm. It can provide quantitative rules that can be interpreted to aid in the analysis. On the Lasso, LightGBM, MCFS, mRMR, and RF feature lists, the optimal DT classifier adopted the first 11,950, 15,650, 20,120, 15,930, and 5,440 gene features. Based on the samples represented by these features, five trees were obtained, from which five groups of classification rules can be extracted. Supplementary Table S4 shows these classification rule groups. Some conditions in major rules would be discussed in detail later.

### 3.4. Enrichment analysis

**Figure 8:**
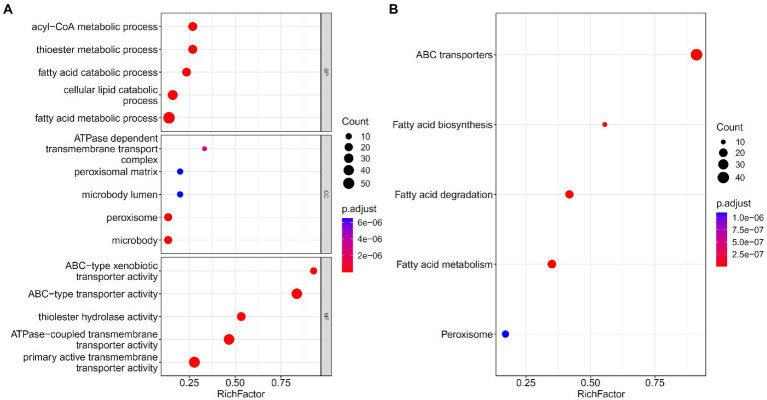
Gene ontology (GO) and KEGG pathway enrichment analysis on the union of five feature sets used to construct feasible random forest classifiers. The FDR < 0.05 criterion was used to filter GO terms and KEGG pathways. The top five significant GO terms in three GO clusters **(A)** and top five KEGG pathways **(B)** were shown.

Five feature sets used to construct five feasible RF classifiers were combined into one set. To uncover the underlying biological meanings behind gene features in such set, the enrichment analysis was conducted on these genes. Figure 8 visualizes top five GO terms in three GO clusters and top five pathways. The GO terms, such as thioester and fatty acid metabolic processes, were enriched, along with peroxisomes and some terms related to metabolism and transport. KEGG enriched pathways included fatty acid biosynthesis, catabolism, and metabolism. Thioesters can be directly involved in the immune response as carriers of antigen presentation and thioesterified fatty acids or other lipid products can be involved in the regulation of immune cells as signaling molecules. Their metabolism is inseparable from the peroxisome.

## 4. Discussion

As listed in “Results”, some essential genes and classification rules were discovered. As they can be strongly related to the response to vaccination in antitumor viral immunity, they were discussed in this section. We collected the scientific findings of other researchers and initially summarized the experimental evidence of the aforementioned genes and rules, proving the accuracy of the findings.

### 4.1. Analysis of essential conditions in rules

Five rule groups were discovered as listed in Supplementary Table S4. As each rule contained multiple gene features and thresholds on expression levels, it was not easy to confirm the special pattern expressed by each rule through existing publications. Thus, we divided each rule into multiple conditions and analyzed the reasonability of some essential conditions. If the conditions used the same gene and same expression trend, they were deemed to be identical. The occurrence number of each condition in five rule groups was counted, which represented how many feature ranking methods identified the condition to be important. Some representative conditions with such numbers larger than two were discussed.

#### 4.1.1. Analysis of conditions identified *via* four methods

*IFI27* occurred in four rule groups, including rule groups on Lasso, LightGBM, mRMR, and MCFS feature lists. The study found that the expression levels of antiviral-related genes such as *IFI27* decreased during the vaccinations. This result is consistent with the dynamically enhanced inflammatory response in vaccinated individuals. *IFI27* is considered a biomarker with high sensitivity and specificity (AUC > 0.85) (Wang et al., 2022). Vaccination can improve the body’s ability to fight viruses. Our analysis results show that the expression level of *IFI27* gradually increased within 2–4 days of the first injection and decreased 7 days after vaccination. However, after the second injection, the expression level of *IFI27* gradually increased within 1–4 days after the injection. Compared with the first injection, some patients had the fastest response times earlier than the first injection. The expression level of *IFI27* decreased 7–10 days after vaccination. The peak duration of the second injection is speculated to be longer than that of the first injection. The antiviral immune-related molecular mechanism of IFI27 has been reported. As a common interferon (IFN)-stimulated gene, *IFI27* encodes a mitochondrial protein that is normally induced by IFN to express and function in most responding cells. It may regulate apoptosis through the stability of mitochondrial membrane, thereby affecting immune response (Cheriyath et al., 2011). In addition, IFI27 can inhibit viral DNA replication and gene expression (Ullah et al., 2021). *In vitro* studies have shown that IFI27 is up-regulated in plasmacytoid dendritic cells, which are antigen-presenting cells sensitive to viral infection (Tang et al., 2017). Transcriptome results showed that vaccinated patients had significantly attenuated IFN responses compared to unvaccinated Omicron and Alpha-infected patients, represented by IFI27, which controls antiviral responses (Lee et al., 2022b). The results of RNA sequencing data analysis showed that macrophages in the blood of SARS-CoV-2-infected patients released a large number of IFNs, activated mitochondrial *IFI27* expression, and disrupted energy metabolism in immune cells, ultimately aggravating viral immune evasion and replication (Duan et al., 2022). Based on existing research reports and our analysis, we speculate that after vaccination, the release of IFN increases, which promotes an increase in mitochondrial protein IFI27, inhibits SARS-CoV-2 replication and gene expression, and enhances antiviral immunity. In addition, after two vaccine doses, some people’s antiviral immunity takes effect earlier than after the first dose, and vaccine efficacy lasts longer. Therefore, *IFI27* may be used as a biomarker for antiviral immunity of vaccines.

#### 4.1.2. Analysis of conditions identified *via* three methods

*Syndecan-1* (*SDC1*) and *small nuclear ribonucleoprotein polypeptide G* (*SNRPG*) were found in rule groups on LightGBM, mRMR, and MCFS feature lists. *SDC1* encodes a transmembrane (type I) heparan sulfate proteoglycan protein that belongs to the syndecan proteoglycan family. As a component of glycocalyx (GAC), SDC1 plays an important role in cell proliferation, cell migration, and other processes through extracellular matrix protein receptors (Reszegi et al., 2022). SDC1 was found to be elevated in COVID-19 patients (Goonewardena et al., 2021). SDC1 may contribute to early risk stratification of staged diseases such as COVID-19 and provide a pathobiological reference (Goonewardena et al., 2021). Studies have confirmed that patients infected with COVID-19 can produce inflammation-induced degradation of the GAC layer of endothelial cells, and SDC1 can be used as an important parameter to assess GAC damage (Vollenberg et al., 2021). High levels of SDC1 may cause more severe endothelial damage and inflammation (Zhang et al., 2021). Molecular experiments demonstrate that *SDC1* acts as a target gene of miR-10a-5p during porcine hemagglutinating encephalomyelitis virus (PHEV) infection and is involved in host defense mechanisms. Decreased expression levels of *SDC1* lead to reduced viral replication, and downstream inhibition of *SDC1* exerts an antiviral effect in PHEV-induced disease (Hu et al., 2020). Transcriptome analysis showed that the expression level of *SDC1* increased only 7 days after the first dose of vaccination. After the second dose, the expression level remained low. On the one hand, this low level may help prevent endothelial damage and severe inflammatory response. On the other hand, it may inhibit viral replication and facilitate a more efficient antibody production.

*SNRPG* is a protein-coding gene involved in the formation of the U1, U2, U4, and U5 small nuclear ribonucleoprotein complexes. Related pathways include SARS-CoV-2 infection and gene expression.^5^ Studies have shown that SNRPG-related risk models are associated with infiltration of immune cells such as T cells and M2 macrophages (Liu et al., 2022). The specific mechanism between *SNRPG* and SARS-CoV-2 infection is limited. Transcriptome analysis showed that the *SNRPG* expression level was high on the day of the first vaccine injection, whereas the expression level was lower on the day of the second vaccine injection. The low *SNRPG* level continued until day 10 after vaccination. The obvious differences in *SNRPG* levels after different injections suggest that the gene can be regarded as an indicator of the effectiveness of vaccination. However, the molecular mechanism needs to be further explored.

#### 4.1.3. Analysis of conditions identified *via* two methods

Rules found in two methods included *TPX2, CCDC28A, FAM227B, PKN2-AS1, NEK2, USP46, C22orf15, SLC20A1, TMSB15A, C2,* and *ZFC3H1*. Some of these genes are associated with antiviral immunity. For example, *TPX2* (microtubule nucleation factor) is a gene whose encoded product is involved in the activation of protein kinase activity, DNA damage, gene transcription, and other physiological processes. PPI network analysis from STRING revealed that as a hub gene, *TPX2* may be a novel COVID-19 intervention target and biomarker (Hasan et al., 2022). As one of the antigen components of a multivalent recombinant fusion protein prophylactic vaccine (rBmHAXT), *TPX2* can promote the production of high titers of antigen-specific antibodies and their isotypes. Animals vaccinated with the *TPX2* antigen secreted higher levels of blood IFN-γ and showed better immune protection compared with unvaccinated animals (Khatri et al., 2018). Studies have shown that *TPX2* can activate Aurora A kinase (AURKA), which is involved in cell cycle regulation. *TPX2* overexpression enhanced cell proliferation and migration (Zou et al., 2018). The *TPX2* gene may be a potential target for diagnosis and prognosis in patients already infected with hepatitis B virus (HVB) (Ji et al., 2020). Transcriptome data analysis showed that *TPX2* expression levels increased within 7–10 days after the patients received the second vaccine dose. This is consistent with activation of IFN-induced responses, increased transcripts of specific IGHV clones, and a trend toward memory B cell enrichment (Lee et al., 2022a). *TPX2* may be related to antiviral immunity caused by different doses. However, the correlation and mechanism of action need to be further verified.

### 4.2. Top features identified *via* multiple methods

On the basis of the features identified by the five feature ranking algorithms (Figure 7), an intersection of results obtained by multiple methods (≥3) was selected as important candidates. We summarized the evidence for some vital gene features, listed in Table 5, based on the broad studies shown below.

**Table 5:** Essential genes identified by three feature ranking algorithms.

Index	Gene symbol	Description
1	RPRD1B	Regulation of nuclear pre-mRNA domain containing 1B
2	NFE2L2	NFE2-like bZip transcription factor 2
3	SMC5	Structural maintenance of chromosome 5
4	NEU3	Neuraminidase 3

*NFE2-like bZip transcription factor 2* (*NRF2*), also called *NFE2L2*, encodes a cap‘n’collar (CNC) transcription factor and belongs to the small family of basic leucine zipper (bZIP) proteins (Khan et al., 2021). *NRF2* can bind to antioxidant response elements and participate in the transcription of downstream target genes. Thus, it plays an important role in physiological processes such as cellular redox, tissue damage, and metabolic homeostasis. The encoded protein of *NRF2* is involved in various injury and inflammatory responses involving class I MHC-mediated antigen presentation and KEAP1-NFE2L2 pathway, among others. *NRF2* contributes to GSH metabolism and stress response and is associated with the pro-inflammatory effects of SARS-CoV-2 in host cells (Galli et al., 2022). The protein synthesis of SARS-CoV-2 may increase Cys and activate endoplasmic reticulum stress of transcription factors, which ultimately promotes changes in cellular oxidation, cellular metabolism, and GSH transmembrane flux (Galli et al., 2022). Importantly, *NRF2* activation has been shown to benefit respiratory infections in various animal models (Muchtaridi et al., 2022). *NRF2* exerts anti-inflammatory effects by inhibiting pro-inflammatory genes such as *IL6* and *IL1B* (Huang et al., 2022). *NRF2* induces the expression of genes that promote specificity of macrophages such as the macrophage receptor, which is responsible for bacterial phagocytosis (Schaefer et al., 2022), and the cluster of differentiation gene 36 (CD36), which resists viral infection (Hillier et al., 2022). *NRF2* Activation is involved in inflammatory cascade (Jayakumar et al., 2022), regulation of innate immune responses, and antiviral cytosolic DNA sensing. *NRF2* inhibits pro-inflammatory signaling pathways such as TNF-α signaling and is involved in regulating the innate immune response during sepsis. *NRF2* increases susceptibility to DNA virus infection by inhibiting the expression of the adaptor protein STING1, thereby inhibiting antiviral cytosolic DNA sensing (Olagnier et al., 2018). After SARS-CoV-2 infection, *NRF2* is activated and restricts the release of pro-inflammatory cytokines by inhibiting IRF3 dimerization. In addition, *NRF2* inhibits the replication of SARS-CoV-2 and other viruses through a type I IFN-independent pathway (Olagnier et al., 2020).

*Regulation of nuclear pre-mRNA domain containing 1B* (*RPRD1B*), also named cell-cycle-related and expression-elevated protein in tumor (*CREPT*) or *C20ORF77*, is located on chromosome 20q11 and can bind to RNA polymerase on the cyclin D1 gene, resulting in the formation of a cyclin D1 ring structure, which can promote transcription (Lu et al., 2012; Wang et al., 2014). *RPRD1B* can also participate in the transcription of genes related to the Wnt/β-catenin signaling pathway (Wu et al., 2010). GO annotation results showed that *RPRD1B* can bind to the RNA polymerase II complex and play a role in pathways such as TCR signaling and T-cell activation. The mRNA and protein expression of *RPRD1*B in patients under 50 years old were significantly different from those in patients over 50 years of age. *RPRD1B* expression levels correlate with human papillomavirus infection and may be affected by age (Wen et al., 2021). The expression level of *RPRD1B* in peripheral blood T cells of psoriasis, lichen planus (LP), and atopic dermatitis (AD) was found higher than that of healthy subjects. *RPRD1B* is involved in the pathogenesis of inflammatory diseases by regulating the transcription of genes such as *IL-4*, *RGS16*, and *CD30* (Li et al., 2013). Our analysis showed that the *RPRD1B* expression level changed in patients who received different vaccinations. Combined with existing evidence, we speculate that *RPRD1B* uses T cells as a carrier to play a role in antiviral immunity.

Neuraminidase 3 (*NEU3*) is a protein-encoding gene whose product is located in the plasma membrane and belongs to the glycohydrolase family. Its activity is specific to gangliosides and may be involved in gangliosides in lipid bilayer adjustment. Pathways associated with *NEU3* include protein metabolism and glycosphingolipid metabolism. It can directly interact with signaling receptors such as EGFR to regulate transmembrane signaling (Wada et al., 2007; Mozzi et al., 2015). Sialidase activity in human polymorphonuclear leukocytes plays a key role in infection and inflammatory responses (Cross et al., 2003; Sakarya et al., 2004). Sialidase activity is determined by membrane-associated sialidase (*NEU3*), which promotes cell adhesion and cell proliferation. Combined with existing evidence, our results indicate that after vaccination, the body produces antibodies against SARS-CoV-2 that regulate the host immune response by affecting the activity of *NEU3*.

The encoded product of structural maintenance of chromosome 5 (*SMC5*) has ATP-binding activity and is involved in physiological processes such as DNA recombination, cellular senescence, protein metabolism, and transport of mature mRNAs. In addition, SMC*5* can bind to SMC6, participate in the repair of DNA double-strand breaks through homologous recombination, and prevent the transcription of free DNA such as circular virus DNA genomes (Decorsière et al., 2016). Proteomic analysis revealed that Epstein–Barr virus infection disrupts the adhesion proteins SMC5/6, thereby affecting DNA damage repair. In the absence of the involucrin protein BNRF1, SMC5/6 interferes with the formation and encapsidation of viral replication compartments (RCs), ultimately affecting viral lytic replication. SMC5/6 may act as intrinsic immunosensors and restriction factors of human herpes virus RC in viral infectious diseases (Yiu et al., 2022). The SMC5/6 complex compresses viral chromatin to silence gene expression; thus, its depletion enhances viral expression. The SMC5/6 complex also functions in immunosurveillance of extrachromosomal DNA (Dupont et al., 2021). As an intrinsic antiviral restriction factor, Smc5/6, when localized to nuclear domain 10 (ND10) in primary human hepatocytes, inhibits HBV transcription without inducing an innate immune response (Niu et al., 2017). We screened SMC5 signatures in populations vaccinated with different doses. The results suggest that SMC5 may serve as an indicator of vaccine effectiveness.

## 5. Conclusion

The purpose of this study was to analyze the blood transcriptome in response to different numbers and timing of vaccinations through a variety of machine learning algorithms. It also aimed to identify antiviral immunity-related molecules in different vaccinated populations. The feature intersection of multiple analysis methods reflects the effects of different vaccinations on host gene expression. The analysis results showed that the key gene features were highly consistent with existing research conclusions, which helped us to further clarify the possible mechanisms of these genes. The important antiviral immune characteristics obtained in this study will help in understanding the differences in mechanisms of action of different vaccinations and provide a reference for targeted COVID-19 intervention and for optimization of vaccine strategies.

## Data availability statement

Publicly available datasets were analyzed in this study. This data can be found here: https://www.ncbi.nlm.nih.gov/geo/query/acc.cgi?acc=GSE201533.

## Author contributions

TH and Y-DC designed the study. JL, WG, and KF performed the experiments. JR and HL analyzed the results. JL, JR, and HL wrote the manuscript. All authors contributed to the research and reviewed the manuscript.

## Funding

This research was supported by the National Key R&D Program of China [2022YFF1203202], Strategic Priority Research Program of Chinese Academy of Sciences [XDA26040304, XDB38050200], the Fund of the Key Laboratory of Tissue Microenvironment and Tumor of Chinese Academy of Sciences [202002], and Shandong Provincial Natural Science Foundation [ZR2022MC072].

## Conflict of interest

The authors declare that the research was conducted in the absence of any commercial or financial relationships that could be construed as a potential conflict of interest.

## Publisher’s note

All claims expressed in this article are solely those of the authors and do not necessarily represent those of their affiliated organizations, or those of the publisher, the editors and the reviewers. Any product that may be evaluated in this article, or claim that may be made by its manufacturer, is not guaranteed or endorsed by the publisher.

## Footnotes

^1^https://lightgbm.readthedocs.io/en/latest/

^2^http://www.ipipan.eu/staff/m.draminski/mcfs.html

^3^http://home.penglab.com/proj/mRMR/

^4^https://github.com/scikit-learn-contrib/imbalanced-learn

^5^https://pathcards.genecards.org/Card/sars-cov-2_infection?queryString=SNRPG

## Supplementary material

SUPPLEMENTARY TABLE S1 Feature lists obtained using Lasso, LightGBM, MCFS, mRMR and PFI.

SUPPLEMENTARY TABLE S2 Performance of IFS with different classification algorithms.

SUPPLEMENTARY TABLE S3 Intersection of feature sets used to construct feasible random forest classifiers on five feature lists.

SUPPLEMENTARY TABLE S4 Classification rules generated by the optimal DT classifier on different feature lists.
